# Dysbaric Osteonecrosis in Divers: A Narrative Review With a Systematic Literature Search of Pathophysiology, Prevalence, Clinical Features, and Screening

**DOI:** 10.7759/cureus.102625

**Published:** 2026-01-30

**Authors:** Rima Houssaini, Yasmina Abdelrazik

**Affiliations:** 1 Medicine, Great Western Hospital Academy, University of Bristol, Bristol, GBR; 2 Ophthalmology, Great Western Hospital, Swindon, GBR

**Keywords:** clinical features, dysbaric osteonecrosis, pathophysiology, prevalence, screening

## Abstract

Dysbaric osteonecrosis (DON) is a form of bone injury that occurs when nitrogen bubbles form during rapid decompression, leading to impaired blood flow and subsequent bone necrosis. Although DON was first described more than a century ago, it remains underdiagnosed, particularly among unregulated diving populations. This review aims to summarize available data on the prevalence, anatomical distribution, clinical presentation, and diagnostic methods of DON in divers, while also identifying research gaps and evaluating proposed pathophysiological mechanisms. A literature search was conducted across Google Scholar, PubMed, Embase, and Scopus. Twelve studies (seven prevalence studies and five case-based reports) were included based on relevance and sample size. Reported prevalence varied widely, with unregulated artisanal divers showing markedly higher rates (up to 76.9%) compared with regulated populations, such as military divers (0%-3.125%). Plain radiography and magnetic resonance imaging (MRI) were the most commonly used diagnostic modalities. DON predominantly involved long bones, particularly the humeral and femoral heads, likely reflecting their terminal blood supply and susceptibility to ischemia. Type B (diaphyseal) lesions were typically incidental, whereas Type A (subchondral) lesions were more often symptomatic. Proposed pathophysiological mechanisms include thrombus formation secondary to adipocyte rupture and direct vascular obstruction by nitrogen bubbles. Although the role of prophylactic anticoagulation remains uncertain, elevated plasminogen activator inhibitor-1 (PAI-1) levels in some cases suggest a coagulative component. Studies relying primarily on radiography reported higher prevalence rates, likely reflecting detection bias rather than true disease burden, given the low sensitivity of X-ray imaging for early DON. MRI, considered the diagnostic gold standard, was predominantly used in controlled or regulated settings. DON is a preventable complication of decompression exposure. Adherence to established diving protocols and early screening using sensitive imaging modalities are essential. Further prospective studies and evaluation of emerging diagnostic tools, such as computed tomography (CT), are needed to improve detection, prevention, and overall diving safety.

## Introduction and background

Decompression sickness (DCS) occurs when inert gas bubbles form in the body during rapid ascent from depth, leading to vascular obstruction and impaired tissue perfusion. DCS is associated with dysbaric osteonecrosis (DON), a form of ischemic bone necrosis that predominantly affects individuals exposed to pressure changes, particularly divers. Radiologically, DON lesions are commonly classified as Type A (subchondral) and Type B (diaphyseal). Although often asymptomatic in its early stages, DON may progress to cause joint pain, restricted mobility, and eventual joint collapse [1].

Despite being recognized for over a century, DON remains frequently overlooked or diagnosed at advanced stages. The earliest cases were reported in 1911 among compressed-air workers and in 1936 in divers [2]. Delayed diagnosis is largely attributable to its insidious clinical course and reliance on imaging modalities such as magnetic resonance imaging (MRI) or plain radiography, a challenge that is particularly pronounced in low-resource settings and among unregulated diver populations.

This literature review addresses three primary objectives: to assess the prevalence of DON across different diving populations; to describe the most commonly affected anatomical sites and associated clinical features; and to evaluate the imaging modalities used for early detection. Additionally, the review examines current hypotheses regarding the pathophysiology of DON and identifies gaps in the existing evidence that limit early diagnosis and prevention. A narrative review approach was selected due to the heterogeneity of available studies, which include observational research and case reports, with limited high-quality comparative data. Clarifying these issues is increasingly important given the growing participation in recreational diving and the continued reliance on commercial and military diving worldwide.

## Review

Method

This review followed the Preferred Reporting Items for Systematic Reviews and Meta-Analyses (PRISMA) principles where applicable, but was not conducted as a formal systematic review. An initial phase of preliminary research involved a broad review of peer-reviewed literature and other credible sources. Google Scholar was used for this initial search, as it allows identification of relevant studies not indexed in traditional databases. Using keywords such as “dysbaric osteonecrosis” and “divers”, two relevant studies were identified at this stage.

**Figure 1:**
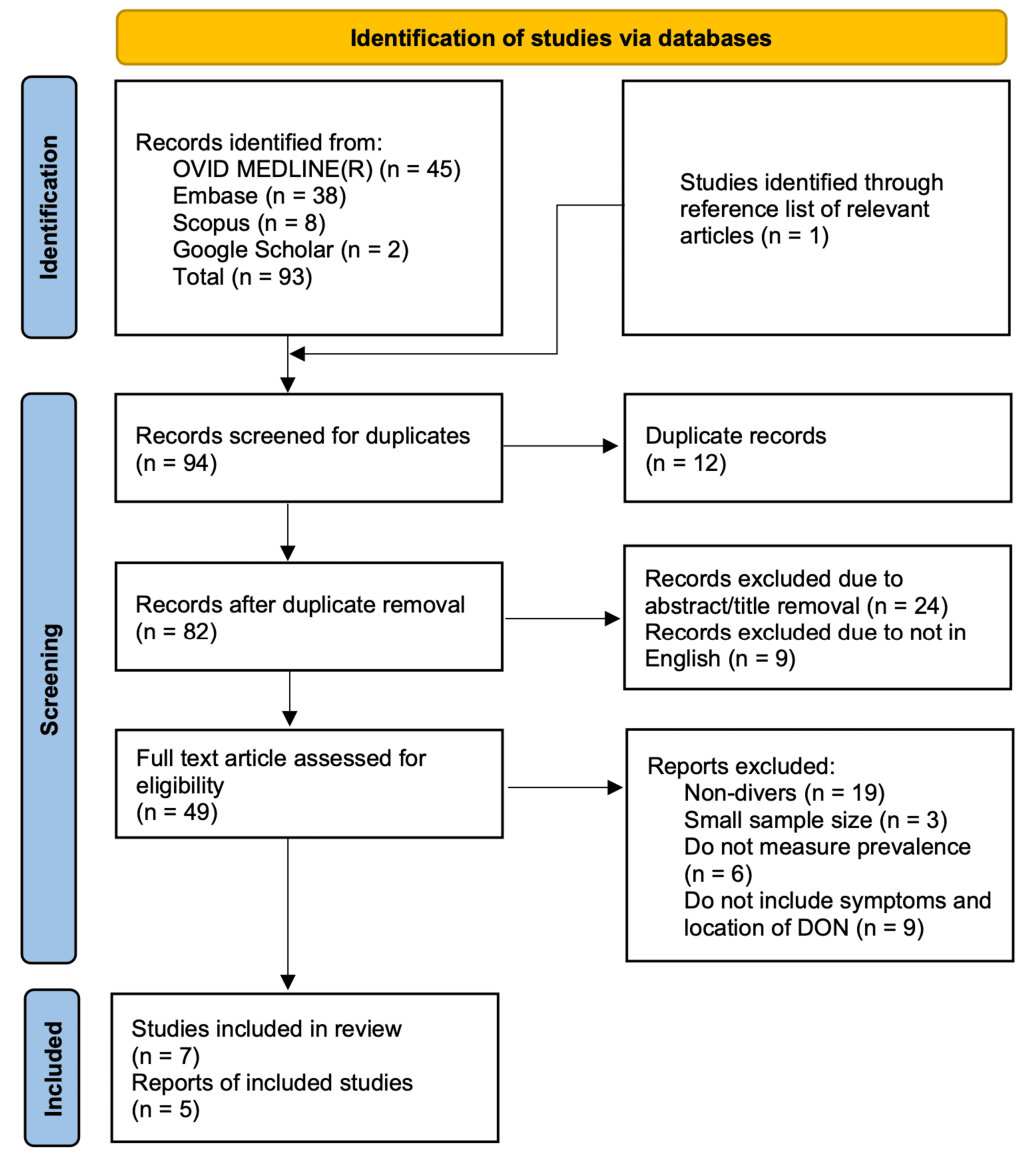
Flowchart of the study selection process.

A more structured literature search was subsequently conducted in June 2025, with the final search completed on June 30, 2025, using three databases: OVID MEDLINE® via PubMed, Embase, and Scopus. An overview of the study selection process is presented in Figure 1.

The search strategy utilized Boolean operators with the following terms: “((dysbaric osteonecrosis) OR (avascular necrosis)) AND ((screening) OR (detection) OR (identif*)) AND ((divers) OR (diving))”. Review of reference lists identified one additional study, yielding a total of 94 records. After removing duplicates using a deduplication tool, 12 studies were excluded, leaving 82 studies for title and abstract screening based on relevance and availability in English, as translating non-English texts was beyond the scope of this review. Of these, 49 studies underwent full-text screening. Inclusion criteria prioritized studies with sample sizes greater than 20 to better reflect the broader diver population and improve generalizability and reliability of findings [3]. Given the limited number of eligible studies identified, case reports were also included. Based on these criteria, 12 studies and case reports were ultimately included in this literature review. 

As this review focused on peer-reviewed literature indexed within selected databases, emerging studies and grey literature may not have been fully captured. A formal risk-of-bias assessment was not performed, as the review was narrative in nature and included heterogeneous study designs. While the methodology is described in sufficient detail to support transparency and interpretation, the review was not intended to allow strict replication.

Due to substantial heterogeneity among the included studies, results were summarized descriptively. This included reporting prevalence ranges, proportions of affected divers, comparisons of anatomical distribution, and differences in imaging modalities used across studies.

Results

Data from the 12 studies (7 prevalence studies and 5 case reports) were extracted, analyzed, and summarized in Table 1 and Table 2.

**Table 1:** Summary of the included studies, highlighting the prevalence of DON among different diver types and which image modality was used. DON: dysbaric osteonecrosis.

Author	Type of study	Location	Type of diver	Number of subjects	Prevalence of DON (%)	Method of detection
Körpınar et al (2021) [4]	Cross-sectional study	Turkey	Recreational diver	46	2.17	MRI
Popa et al (2020) [5]	Cross-sectional study	Mexico	Diving fisherman	39	76.9	X-ray
Gempp et al (2009) [6]	Observational retrospective	France	Recreational divers	288	2.08	MRI
Uzun et al (2008) [7]	Cross-sectional study	Turkey	Navy divers	106	0	MRI
Cimsit et al (2007) [8]	Cross-sectional study	Turkey	Dive instructors	56	25	X-ray
Miyanishi et al (2006) [9]	Cross-sectional study	Japan	Diving fisherman	56	55	X-ray
Bolte et al (2005) [10]	Controlled cross-sectional study	Germany	Military divers	32	3.125	MRI

**Table 2:** Summary of the case reports, highlighting the symptoms, location, and duration of DON among different diver types. DON: dysbaric osteonecrosis, N/A: not applicable.

Author	Type of diver(s)	Depth of dive	Location of DON	Symptoms experienced	Duration of symptoms	Method of detection	Additional method of detection
Kurtul and Güngördü (2022) [11]	Diving fisherman	20-25 m	Left shoulder	Initially: widespread pain. Delayed: left shoulder pain, left arm weakness	1-2 years	X-ray	MRI
Jitsuiki et al (2021) [12]	Recreational diver	26 m	Left hip, right knee, bilateral shoulder joints, and right intramedullary humerus	Headache, general fatigue, pain in both shoulders and elbows	205 minutes	CT	N/A
Briceño-Souza et al (2019) [13]	Diving fisherman	30 m	Left hip	Pain in the left hip, inguinal pain, paresthesia, decreased mobility	8 years	X-ray	CT
Stéphant et al.(2008) [14]	Recreational divers	45 m	Diver 1: right shoulder; diver 2: left elbow and left shoulder	Pain in right shoulder: 1 diver; pain in left elbow and left shoulder: 1 diver	48 hours	MRI	N/A
Wilmshurst and Ross (1998) [15]	Recreational divers	40 m	Right shoulder	Increasing discomfort and restricted movement	18 months	X-ray	MRI

Prevalence of DON

As provided in Table 1, there were notable differences in the prevalence of DON between studies.

Among recreational divers in Turkey, one study reported a low prevalence of DON (2.17%), detected using MRI [4]. This closely aligns with findings from a study of French recreational divers, which reported a prevalence of 2.08% [6]. These low prevalence rates contrast sharply with those observed among diving fishermen. The three highest reported prevalences of DON (76.9%, 55%, and 25%) were identified using X-ray imaging [5,8,9]. In contrast, one study reported a prevalence of 0% among Turkish Navy divers [7], while another observed a prevalence of 3.125% in German military divers [10]. Overall, these findings indicate that DON prevalence is lowest in regulated diving populations, such as military divers, and highest in unregulated settings, particularly among diving fishermen.

Clinical Presentation of DON

As described in Table 2, a wide range of DON symptoms, from mild discomfort to long-term functional impairment, are revealed by the case studies.

One recurrent issue was long-term pain. For example, one case report described shoulder symptoms that developed and persisted for over 18 months, while another reported hip and groin pain with associated limited mobility lasting up to eight years [13,15]. In contrast, two case reports described symptom resolution within 205 minutes and 48 hours, respectively [12,14]. Additionally, two separate case reports documented neurological symptoms, including paresthesia and arm weakness [11,13].

Location of DON

Table 2 highlights that DON predominantly affects long bones and large joints. Across the five case reports, the shoulder joint, particularly the humeral head, emerged as the most frequently affected site, appearing in four of the five reports. The hip joint was another commonly involved location, reported in two cases. Additional sites included the elbow, knee, and intramedullary humerus, reflecting a predilection for large, weight-bearing, or highly mobile joints.

Method of Detection

Based on Table 1 and Table 2, MRI was the most commonly used screening modality, appearing in four prevalence studies and three case reports. Notably, MRI was primarily utilized in regulated populations, such as recreational and military divers. In contrast, X-ray imaging was used more frequently in unregulated groups, particularly diving fishermen. Computed tomography (CT) was the least commonly used modality, appearing only in two case reports and in none of the prevalence studies.

Discussion

Pathophysiology

The pathophysiology of DON is primarily attributed to ischemic bone injury resulting from reduced blood flow [1]. This reduction is thought to arise from nitrogen bubble formation during decompression, particularly when ascent occurs too rapidly [16].

This mechanism is closely linked to Henry’s Law, which states that the amount of gas dissolved in a liquid increases with pressure [17]. During descent, increasing ambient pressure leads to greater dissolution of nitrogen into the blood and tissues. If ascent is rapid, nitrogen cannot be adequately eliminated and instead forms intravascular bubbles. These bubbles may obstruct blood vessels, impairing perfusion to bone and resulting in ischemia [1]. Standard decompression practices are therefore emphasized to reduce risk; for example, recreational dives commonly include a three-minute safety stop to allow sufficient nitrogen off-gassing and promote safe ascent [18].

An alternative hypothesis proposes that nitrogen bubbles may contribute to DON not only by vascular obstruction but also through direct injury to the bone marrow. Experimental models and human autopsy studies have demonstrated the formation of nitrogen bubbles within fatty bone marrow following rapid decompression [19]. Expansion of these bubbles may exert mechanical stress on marrow adipocytes, disrupt the surrounding microvasculature, impair local blood flow, and promote thrombus formation, ultimately contributing to osteonecrosis.

Supporting this theory, one study reported elevated levels of PAI-1, a marker of impaired fibrinolysis, in 31 of 56 divers with DON [9]. While this finding suggests a possible prothrombotic component, the association remains observational. Consequently, anticoagulant therapy has been proposed as a potential preventive strategy rather than an established intervention.

Although both models converge on compromised bone perfusion as the final pathway, the adipocyte rupture hypothesis introduces a thrombotic mechanism not accounted for in the classical intravascular bubble theory. Confidence in its generalizability remains limited by the absence of interventional trials evaluating anticoagulant use.

Clinical Presentation

DON predominantly affects the long bones, reflecting its underlying pathophysiology of ischemic bone injury due to impaired blood supply. Radiologically, DON lesions are classified into two types: Type A lesions, which are subchondral and often symptomatic, and Type B lesions, which are diaphyseal and usually asymptomatic [9]. Type A lesions, particularly those involving the femoral and humeral heads, are more vulnerable because of their limited vascular supply, making them more likely to become symptomatic and increasing the risk of joint collapse [1].

Clinically, DON most commonly presents with joint pain, stiffness, or reduced mobility, especially in large weight-bearing or highly mobile joints such as the hip and shoulder. In more advanced cases, symptoms may extend to neurological features including paresthesia and limb weakness, as reported in two studies, possibly reflecting more severe ischemia or neural involvement [11,13]. Although case reports are inherently anecdotal and may over-represent severe disease, they consistently reinforce patterns observed in larger studies, particularly the predilection for shoulder and hip involvement.

Screening

Screening for DON can be challenging because the condition is often asymptomatic in its early stages and diagnosis relies heavily on imaging. From the available literature, plain radiography remains the most commonly used screening tool, particularly in occupational divers, such as fishermen, largely due to its low cost, wide availability, and rapid acquisition [20]. However, X-ray has low sensitivity for early or asymptomatic disease and therefore tends to detect only advanced-stage lesions [21]. In one study, X-ray was used as the sole screening modality and reported a high prevalence of 76.9%, likely reflecting detection of late-stage, symptomatic DON rather than true disease burden [5]. The unregulated nature of artisanal diving may further contribute to these high prevalence rates. The absence of an MRI comparison in this study represents an important limitation.

In contrast, MRI is considered the gold standard for DON screening because of its high sensitivity and ability to detect early and asymptomatic lesions [22]. Nevertheless, its use is constrained by high cost, limited availability, and the potential for false-positive findings, which can complicate clinical decision-making [4]. Although MRI-based studies often report lower prevalence rates than X-ray studies, this may reflect greater diagnostic specificity rather than a true reduction in disease occurrence [23]. Most MRI studies, however, involved sample sizes of fewer than 100 participants, limiting the precision and generalizability of prevalence estimates and underscoring the need for larger studies.

CT offers another potential imaging modality, providing high sensitivity and specificity for detecting small osseous lesions suggestive of DON [24]. However, CT was utilized in only two case reports, and the available evidence remains insufficient to draw firm conclusions regarding its role in screening or diagnosis. Further research is needed to clarify whether CT may serve as a useful adjunct or alternative to MRI, particularly in settings where MRI access is limited.

Limitations

There are several important gaps in the current evidence on DON. Prospective cohort studies are largely lacking, which limits understanding of disease progression and the natural history of DON. Cross-sectional studies can estimate prevalence but cannot determine causation or track lesion development over time, and they may miss cases due to survivor bias. Retrospective studies, meanwhile, depend on historical records, which can introduce selection bias and lead to underreporting. This literature review is also subject to potential publication bias and did not include a formal meta-analysis. Moreover, recently published or non-indexed studies may have been missed despite comprehensive search efforts.

## Conclusions

DON remains a clinically important yet frequently overlooked complication of diving. This review found that DON predominantly affects the long bones, which aligns with its ischemic pathophysiology and vascular vulnerability. Type A lesions were more likely to be symptomatic and detected in imaging, while Type B lesions were often asymptomatic. The rates of DON varied between different groups of divers. The highest rates were observed in unregulated divers, like artisanal fishermen, while military divers had the lowest rates. This shows how important training, decompression procedures, and good screening methods are to reduce the risk. Screening also varied: X-rays are easier to use but lack sensitivity, while MRIs are better at finding asymptomatic lesions but are more expensive and less available.

Future research should include prospective cohort studies using CT alongside MRI to assess early lesion detection rates, particularly in different diver populations. Additionally, studies should concentrate on clarifying the role of prothrombotic factors, such as PAI-1. Future literature reviews would be improved by the establishment of a universal DON classification system that considers lesion site, type, and symptoms. In conclusion, early detection and prevention are essential to reduce prevalence and to ultimately ensure safer diving practices across all populations.
